# *DRD2* Ex8 rs6276 Polymorphism and NEO-FFI Personality Traits in Elite Athletes and Controls

**DOI:** 10.3390/brainsci15090965

**Published:** 2025-09-05

**Authors:** Remigiusz Recław, Milena Lachowicz, Jolanta Chmielowiec, Dariusz Larysz, Anna Grzywacz, Krzysztof Chmielowiec

**Affiliations:** 1 Independent Laboratory of Behavioral Genetics and Epigenetics, Pomeranian Medical University in Szczecin, Powstancow Wielkopolskich 72 St., 70-111 Szczecin, Poland; remigiusz.reclaw@pum.edu.pl; 2 Department of Medical Sciences and Public Health, Gdansk University of Physical Education and Sport, Kazimierza Gorskiego 1 St., 80-336 Gdansk, Poland; 3 Department and Clinic of Oncology and Radiotherapy, Medical University of Gdansk, ul. M. Skłodowskiej-Curie 3a, 80-210 Gdansk, Poland; milena.lachowicz@awf.gda.pl; 4 Department of Psychology, Gdansk University of Physical Education and Sport, Kazimierza Górskiego 1 St., 80-336 Gdansk, Poland; 5 Department of Hygiene and Epidemiology, Collegium Medicum, University of Zielona Góra, 28 Zyty St., 65-046 Zielona Gora, Poland; chmiele1@o2.pl (J.C.); chmiele@vp.pl (K.C.); 6 109th Military Hospital with Polyclinic, Ministry of National Defense, ul. Ksiedza Piotra Skargi 9/11, 71-422 Szczecin, Poland; dariuszlarysz@hotmail.com

**Keywords:** *DRD2* Ex8 rs6276, dopamine D2 receptor, NEO-FFI, professional athletes, genetic polymorphism, personality and motivation, behavioral genetics

## Abstract

**Background/Objectives:** Personality traits influence motivation, self-regulation, and adaptation in high-performance sports, and are partially modulated by dopaminergic genetic variability. This study aimed to examine the association between the *DRD2* Ex8 rs6276 polymorphism and NEO Five-Factor Inventory (NEO-FFI) personality traits in elite athletes and non-athlete controls. **Methods:** A total of 323 participants were included: 141 athletes and 182 controls. Genomic DNA was isolated from venous blood, and *DRD2* Ex8 rs6276 genotypes (A/A, A/G, G/G) were determined using real-time PCR with melting-curve analysis. Personality traits were assessed using the NEO-FFI, and group differences as well as genotype × group interactions were evaluated using multivariate analyses and non-parametric tests. **Results:** Athletes scored significantly higher on Conscientiousness than controls. A genotype × group interaction was observed for Extraversion, and the main effect of the genotype was found to be Agreeableness. Athletes with the A/A genotype exhibited the highest Extraversion scores, whereas those with the G/G genotype demonstrated higher Agreeableness than other genotypes. **Conclusions:** These findings indicate that dopaminergic variation contributes to individual differences in social and motivational traits, which may support athletic engagement and adaptation to high-demand environments. The results should be interpreted with caution due to the moderate sample size, deviation from the Hardy–Weinberg equilibrium in the athlete group, and reliance on a single personality assessment tool.

## 1. Introduction

Dopaminergic signaling plays a fundamental role in regulating motivation, reward processing, and goal-directed behavior. The dopamine D2 receptor (*DRD2*) is a key component of the mesolimbic reward pathway, which is implicated in addictive behaviors, impulse control, and reinforcement learning [1,2,3]. Variants of the *DRD2* gene, including single-nucleotide polymorphisms (SNPs), such as Ex8 rs6276, have been linked to individual differences in reward sensitivity, vulnerability to substance use disorders, and motivational traits [4,5,6].

The *DRD2* gene, located on chromosome 11q23.2, encodes the dopamine D2 receptor, which plays a key role in striatal reward processing and behavioral control [7]. The Ex8 rs6276 variant is a synonymous SNP that, despite not altering the amino acid sequence, may affect mRNA stability and receptor expression, potentially influencing reward sensitivity and impulsivity [4,8,9]. Despite its functional relevance, rs6276 has rarely been examined in the context of high-performance sport.

Personality traits assessed with the NEO-FFI are linked to dopaminergic function and behavioral regulation. High Extraversion and low Conscientiousness are associated with risk-taking and impulsivity, whereas high Conscientiousness supports self-discipline and long-term goal pursuit, which are crucial for elite sports performance [10,11]. Previous research has reported inconsistent findings linking *DRD2* polymorphisms with personality traits such as Extraversion, Conscientiousness, and Neuroticism [12,13,14,15,16,17], with discrepancies often attributed to small sample sizes, heterogeneous populations, and methodological variation.

Professional athletes represent a unique population for studying gene–personality associations, given their long-term exposure to structured training, motivational demands, and stress stimuli [18]. Understanding how dopaminergic polymorphisms interact with personality traits in this group may help optimize training strategies, identify vulnerability to maladaptive coping, and inform personalized psychological support.

Study aims and hypothesis: The present study aimed to examine the association between the *DRD2* Ex8 rs6276 polymorphism and NEO-FFI personality traits in elite athletes compared with non-athlete controls. The study hypothesizes that rs6276 could be associated with Extraversion and Conscientiousness, with effects differing between athletes and controls.

## 2. Materials and Methods

### 2.1. Materials

The study involved a total of 323 volunteers, including 141 professional athletes (M = 92, 65%; F = 49, 35%; mean age = 22.7, SD = 6.02) and 182 non-addicted control participants (M = 146, 80%; F = 36, 20%; mean age = 21.9, SD = 3.88). The control group consisted mainly of university students and young adults recruited from the local community through advertisements. Participants were age-matched to the athlete group, and individuals with a history of substance dependence or psychiatric disorders were excluded on the basis of the MINI. Sex differences were examined, but no significant effects were found; therefore, sex was not included as a covariate in the final models. The research protocol was reviewed and approved by the Bioethics Committee for Clinical Research of the Regional Medical Society in Szczecin (Marii Skłodowskiej-Curie 11 Street, protocol no. 13/KB/VI/2016, 8 December 2016). All participants provided written informed consent prior to inclusion in the study [19].

The project was conducted at the Independent Laboratory of Health Promotion. Both the professional athletes and the control group underwent psychiatric assessment using the Mini International Neuropsychiatric Interview (MINI) and the NEO Five-Factor Personality Inventory (NEO-FFI).

The athletes were active competitors in national or international sports events, representing various disciplines: karate (n = 6), judo (n = 17), boxing (n = 19), MMA (n = 31), ju-jitsu (n = 5), kickboxing (n = 8), volleyball (n = 5), handball league (n = 5), ice hockey (n = 24), triathlon (n = 1), basketball (n = 4), and football (n = 16). All professional sportsmen and sportswomen had participated in competitions within the year preceding the study and had been consistently training for at least five years [19]. Due to the heterogeneity of sports disciplines, exploratory subgroup comparisons (combat vs. team sports) were attempted; however, the sample sizes were insufficient to draw robust conclusions.

The study focused on assessing the interaction between personality traits and the *DRD2* Ex8 rs6276 gene polymorphism in both athletes and control participants.

### 2.2. Measures

The Mini International Neuropsychiatric Interview (MINI) is a structured diagnostic tool designed to assess psychiatric disorders according to the DSM-IV and ICD-10 criteria.

Personality traits were evaluated using the NEO Five-Factor Inventory (NEO-FFI), which assesses five major domains of personality. Each trait is represented by six specific facets:

- Neuroticism—anxiety, hostility, depression, self-consciousness, impulsivity, and vulnerability to stress;
- Extraversion—warmth, sociability, assertiveness, activity, sensation-seeking, and positive emotions;
- Openness to experience—imagination, aesthetic sensitivity, emotional awareness, curiosity for new ideas, values, and actions;
- Agreeableness—trust, straightforwardness, altruism, compliance, modesty, and tenderness;
- Conscientiousness—competence, orderliness, sense of duty, achievement striving, self-discipline, and deliberation.

Scores from the NEO-FFI were converted into sten scores in accordance with Polish adult normative data. The following interpretation was applied: 1–2 sten—very low results, 3–4 sten—low, 5–6 sten—average, 7–8 sten—high, 9–10 sten—very high [19].

### 2.3. Genotyping

Genomic DNA was extracted from venous blood samples using a commercially available protocol (QIAamp Blood DNA Mini Kit, QIAGEN, Hilden, Germany). Genotyping of the *DRD2* Ex8 rs6276 variant was performed using a real-time PCR assay, following protocols previously described in the literature [19]. During the analysis, the fluorescence signal was monitored and plotted as a function of temperature, producing melting curves for each sample. The allele-specific melting peaks for the *DRD2* Ex8 rs6276 (1800498) variant were detected at 59.1 °C for the G allele and 67.7 °C for the A allele, which allowed precise determination of individual genotypes.

### 2.4. Statistical Analysis

The genotype frequency distribution for the *DRD2* Ex8 rs6276 polymorphism was evaluated for the Hardy–Weinberg equilibrium (HWE) using the HWE software (https://wpcalc.com/en/equilibrium-hardy-weinberg/, accessed on 12 December 2024).

Associations between *DRD2* Ex8 rs6276 genotypes, group status (athletes vs. controls), and the five NEO-FFI personality scales as dependent variables were analyzed using a multivariate factorial ANOVA, including the main effects of genotype and group as well as the genotype × group interaction.

Prior to conducting ANOVA, homogeneity of variance was verified using the Levene test (*p* > 0.05). As the analyzed variables were not normally distributed, Mann–Whitney U tests were additionally applied to compare NEO-FFI dimensions (Neuroticism, Extraversion, Openness, Agreeableness, and Conscientiousness) between athletes and controls. Effect sizes (η^2^) were calculated for each analysis, and where relevant, 95% confidence intervals were reported.

Differences in *DRD2* Ex8 rs6276 genotype frequencies between groups were evaluated using the chi-square test. For these variables, the accepted significance level was 0.01 (0.05/5), using the Bonferroni correction for multiple comparisons. All statistical procedures were performed with STATISTICA 13 (Tibco Software Inc., Palo Alto, CA, USA) for Windows (Microsoft Corporation, Redmond, WA, USA) [19].

## 3. Results

The genotype frequency distribution in the athlete group deviated from the Hardy–Weinberg equilibrium (HWE), whereas the control group showed full compliance with HWE (Table 1).

No statistically significant differences were observed in the distribution of *DRD2* Ex8 rs6276 genotypes between the athlete group and the controls (A/G: 0.35 vs. 0.45; A/A: 0.44 vs. 0.41; G/G: 0.21 vs. 0.14; χ^2^ = 3.838, *p* = 0.1468). Likewise, no statistically significant differences between males and females were found in the frequency of the *DRD2* Ex8 rs6276 polymorphism or in NEO-FFI personality traits, either in the athlete or control groups. Similarly, allele frequencies for the *DRD2* Ex8 rs6276 variant did not differ significantly between the groups (A: 0.62 vs. 0.63; G: 0.38 vs. 0.37; χ^2^ = 0.1495, *p* = 0.6990) (Table 2).

A comparison of different genetic association models of the *DRD2* Ex8 rs6276 polymorphisms, such as codominant; dominant; overdominant; and recessive was performed between the athlete group and the controls, and no statistically significant influences were found (Supplementary Table S1).

The mean values and standard deviations for all NEO-FFI personality dimensions in both athletes and control participants are summarized in Table 3.

Athletes scored significantly higher than controls on the NEO-FFI Conscientiousness scale (7.08 vs. 6.10; Z = 4.031; *p* = 0.0001) (Table 3). The outcomes of the 2 × 3 factorial ANOVA performed for the NEO-FFI sten scores are presented in Table 4.

### 3.1. Extraversion (Sten Scale)

**Figure 1:**
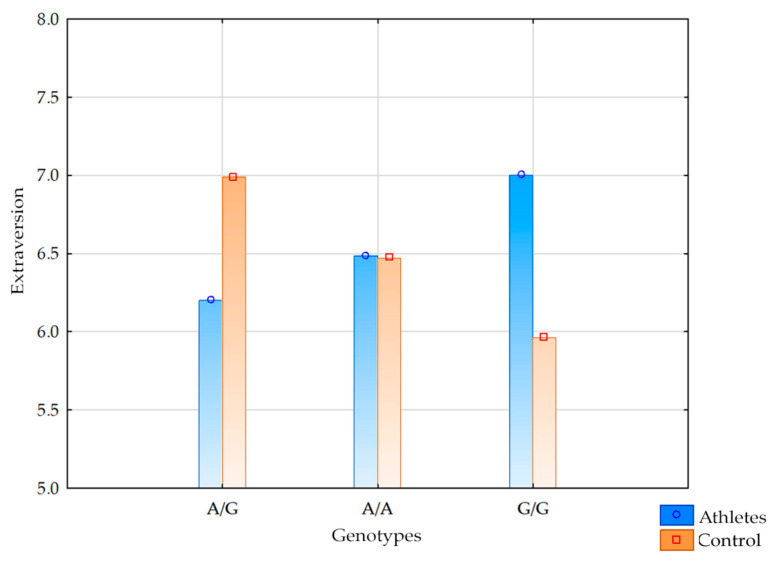
Interaction between group status (athletes vs. controls) and *DRD2* Ex8 rs6276 genotypes for Extraversion (NEO-FFI sten scores). Values represent means ± SE. The interaction effect was significant (F_2,317_ = 5.13, *p* = 0.0064).

A statistically significant interaction was observed between the *DRD2* Ex8 rs6276 genotype and group status (athletes vs. controls) on the Extraversion score (F_2,317_ = 5.13; *p* = 0.0064; η^2^ = 0.031; Figure 1). The observed power for this factor was 82%, and approximately 3% of the variance in Extraversion scores was explained by the interaction between the *DRD2* Ex8 rs6276 polymorphism and group membership.

The post hoc analysis (Table 5) revealed the following:

- Athletes carrying the A/A genotype had significantly higher Extraversion scores than controls with the G/G genotype;
- Athletes with the A/G genotype displayed significantly lower Extraversion scores than controls with the A/G genotype;
- Controls with the A/G genotype showed significantly higher Extraversion scores compared to controls with G/G.

### 3.2. Agreeableness (Sten Scale)

A significant main effect of the *DRD2* Ex8 rs6276 genotype was observed for Agreeableness scores (F_2,317_ = 3.88; *p* = 0.0216; η^2^ = 0.024). The observed power for this factor was 70%, and the genotype accounted for approximately 2% of the variance in Agreeableness scores.

Post hoc comparisons (Table 5) showed the following:

- Athletes with the G/G genotype had significantly higher Agreeableness scores than athletes carrying A/A or A/G;
- Athletes with G/G also scored higher than controls with A/A or A/G genotypes.

### 3.3. Conscientiousness (Sten Scale)

A statistically significant difference in Conscientiousness scores was found between athletes and controls (F_1,317_ = 16.00; *p* = 0.0001; η^2^ = 0.048). The observed power was 98%, and approximately 5% of the variance in Conscientiousness scores was explained by group membership.

The following is true according to the post hoc test (Table 5):

- Athletes with A/A and G/G genotypes demonstrated significantly higher Conscientiousness scores than controls with any genotype (A/A, A/G, or G/G).
- Athletes carrying the A/G genotype also scored significantly higher than controls with the G/G genotype. For clarity, the results are presented as mean values with error bars in Figure 1 (bar plot). To illustrate the interaction pattern more explicitly, an additional line plot with standard errors is provided in the Supplementary Materials (Figure S1).

## 4. Discussion

The present study examined the relationship between the *DRD2* Ex8 rs6276 polymorphism and NEO-FFI personality traits in professional athletes compared with non-athlete controls. The key findings can be summarized as follows:

- No significant differences were found in the distribution of *DRD2* Ex8 rs6276 genotypes, and alleles were observed between athletes and controls.
- Athletes scored higher on the Conscientiousness scale of the NEO-FFI, confirming the importance of self-discipline and goal-directed behavior in competitive sports.
- A significant genotype × group interaction was observed for Extraversion, and a main effect of genotype was observed for Agreeableness, suggesting that dopaminergic variability modulates social and motivational traits differently in athletes and non-athletes.

As shown in Table 3 and Table 4, the group effect for Conscientiousness and the genotype × group interaction for Extraversion remained significant under the Bonferroni-adjusted family-wise threshold (α = 0.01), whereas the main genotype effect for Agreeableness was only nominally significant (*p* = 0.0216) and should be interpreted cautiously.

### 4.1. Personality Traits in Athletes and Their Functional Relevance

The higher Conscientiousness scores observed in athletes align with previous research emphasizing that self-discipline, persistence, and adherence to structured routines are essential traits for long-term success in elite sports [20]. Elevated Conscientiousness has also been associated with better stress regulation, lower impulsivity, and enhanced goal maintenance, which may contribute to sustained athletic engagement and reduced vulnerability to maladaptive behaviors [21,22].

The interaction between Extraversion and the *DRD2* genotype indicates that dopaminergic variation may influence social behavior and reward sensitivity in context-dependent ways. Extraversion has been linked to dopaminergic activity in the mesolimbic pathway, particularly in the striatum, which mediates reward anticipation and approach behavior [23,24]. In this study, athletes with the A/A genotype exhibited higher Extraversion scores, which may reflect greater social engagement and motivational drive—traits that are particularly beneficial in high-intensity competitive environments. Conversely, athletes with the A/G genotype demonstrated lower Extraversion, which may suggest context-specific modulation of social and reward sensitivity by *DRD2* Ex8 rs6276.

Moreover, athletes with the G/G genotype demonstrated higher Agreeableness scores compared to other genotypes, suggesting a possible link between dopaminergic modulation and prosocial or cooperative tendencies in competitive settings.

### 4.2. Dopaminergic Genetics and Behavioral Modulation

Personality traits and motivational dispositions represent complex phenotypes shaped by polygenic and multifactorial influences [10,18,25]. This underscores the fact that the contribution of a single polymorphism, such as *DRD2* Ex8 rs6276, is necessarily limited and should be interpreted within a broader polygenic framework. Dopaminergic signaling is one of the key neurobiological pathways contributing to these traits, but no single polymorphism can fully explain individual variability. Instead, personality emerges from the interplay of multiple genes, their regulatory variants, and environmental exposures such as stress, training, and social context [6,16].

In molecular genetic studies of behavior, it is common to examine single-nucleotide polymorphisms (SNPs) individually. This reductionist approach reflects both methodological constraints and the need to map discrete genotype–phenotype associations within a highly polygenic architecture. Investigating SNPs one by one allows researchers to identify potential functional correlations with personality traits or other dopaminergically linked behaviors, which can subsequently inform larger polygenic or gene × environment interaction models [26].

This study illustrates this stepwise approach. By focusing on the *DRD2* Ex8 rs6276 variant, this study captures a fragment of the dopaminergic contribution to personality. This variant, though synonymous, may influence mRNA stability and receptor expression, subtly modulating dopaminergic neurotransmission [8,9]. In structured, high-demand environments such as elite sports, these small genetic effects can become more apparent, as athletes’ behavioral regulation and reward sensitivity are under constant selective pressure [11].

Taken together, the results are consistent with the broader literature indicating that dopaminergic signaling variants contribute to overlapping personality dimensions such as Conscientiousness and Extraversion [12,13,14,15]. These associations highlight the incremental nature of behavioral genetics research: while personality traits are polygenic and environmentally modulated, examining specific SNP–trait correlations remains a crucial step toward understanding the complex genetic architecture of motivation, self-regulation, and social behavior in elite athletes. At the same time, the effects observed in this study accounted for only a small proportion of variance, underscoring the need for cautious interpretation and for integrating these findings within broader polygenic and environmental frameworks. Although the effect sizes were modest, they may still hold practical significance in the high-demand context of elite sports, where even small differences in motivational or social traits can influence adaptation and performance.

### 4.3. Implications for Sports and Behavioral Genetics

The findings align with previous research suggesting that dopaminergic gene variants contribute to individual differences in personality traits relevant to high-performance sports. Elevated Conscientiousness among athletes in this study is consistent with earlier reports indicating that this trait facilitates long-term adherence to structured training and resilience to stress in elite competitors [20].

The observed interaction between *DRD2* Ex8 rs6276 and athletic status for Extraversion has parallels with studies linking dopaminergic variability to approach-related behaviors and reward sensitivity [12,16]. Specifically, the higher Extraversion scores among A/A carriers in the athlete group may reflect enhanced social engagement and motivational drive, which are traits that can support performance under high demand, whereas reduced Extraversion in A/G carriers may echo reports of allele-specific modulation of social reward processing [13].

Interestingly, the main effect of *DRD2* genotype on Agreeableness resonates with studies that link dopaminergic signaling to prosocial orientation and cooperation [26]. However, this effect did not survive Bonferroni correction across the five NEO-FFI traits and should therefore be regarded as exploratory. These findings suggest that dopaminergic polymorphisms may shape social behavior in ways that are context-dependent: traits that support team functioning and interpersonal stability in sports could, in adverse environments, increase susceptibility to social withdrawal or maladaptive coping. It is also important to note that personality assessment in this study relied solely on the NEO-FFI, which, while well validated, may not capture dimensions such as impulsivity or reward sensitivity that are particularly relevant in sports contexts.

From a broader behavioral genetics perspective, the results support the view that single-gene associations are only fragments of a highly polygenic and environmentally modulated architecture [10]. *DRD2* polymorphisms do not determine behavior in isolation but contribute incrementally to motivational and social tendencies that become most evident in structured, high-pressure contexts such as elite athletics. To evaluate robustness across inheritance assumptions, we additionally examined dominant, recessive, overdominant, and codominant models, which yielded no significant associations (Supplementary Table S1). Future work should integrate polygenic profiles and longitudinal monitoring to clarify how dopaminergic variability interacts with training, stress exposure, and environmental reinforcement to shape both adaptive and maladaptive outcomes in athletes. Importantly, these findings are partially consistent with the initial hypothesis: a genotype × group interaction emerged for Extraversion, whereas for Conscientiousness, only a robust group difference (athletes > controls) was observed without genotype effects, and the main genotype effect for Agreeableness remained nominal after correction.

Beyond the interpretation of personality trait associations, several methodological issues also warrant consideration. It should be noted that the genotype distribution in the athlete group deviated from the Hardy–Weinberg equilibrium (*p* = 0.003). This deviation is likely attributable to the selective nature of the athlete cohort and the moderate sample size rather than genotyping errors, as the control group conformed to HWE. Such deviations are not uncommon in non-random, highly selected populations and should be interpreted with caution in the context of behavioral genetics studies. Moreover, since all samples were genotyped in the same laboratory and the control group showed no deviation from HWE, methodological errors in genotyping can be confidently excluded as an explanation. Although genetic testing is not suitable for direct use in sports career guidance, insights into dopaminergic modulation of personality may support the development of personalized psychological assistance and tailored training programs in elite athletes. Such applications, however, must remain cautious and embedded within a polygenic framework that recognizes the complexity of behavioral traits. At the same time, additional factors such as population stratification or assortative mating represent plausible alternative explanations and should be explicitly considered, which further underscores the need for cautious interpretation of results obtained in highly selected populations such as elite athletes.

### 4.4. Limitations and Future Directions

Several limitations of this study should be acknowledged.

- The sample size was moderate, which limited the statistical power to detect subtle genotype effects or interactions, particularly in genotype × group comparisons.
- Across the five NEO-FFI traits, we applied a Bonferroni-adjusted family-wise threshold of α = 0.01 (0.05/5). Nevertheless, residual multiplicity remains (e.g., for interaction terms and post hoc comparisons), and nominally significant results—such as the main genotype effect for Agreeableness (*p* = 0.0216)—should be interpreted as exploratory.
- The cross-sectional design does not allow causal inference regarding the directionality of gene–behavior associations.
- This study analyzed only a single polymorphism (*DRD2* Ex8 rs6276). Although the research program includes complementary analyses of other *DRD2* and *ANKK1* variants in the same and different athlete cohorts, a fully integrated polygenic approach was not applied here. Future studies should combine multiple dopaminergic polymorphisms in a single model to better capture the genetic architecture of personality and motivational traits.
- The athlete group deviated from the Hardy–Weinberg equilibrium, which likely reflects the selective nature of this cohort and moderate sample size rather than genotyping errors. We also examined dominant, recessive, overdominant, and codominant models, which yielded no significant associations (Supplementary Table S1). Future studies with larger samples may revisit these models to assess robustness under HWE deviations.
- While personality traits were assessed using the NEO-FFI, complementary measures of impulsivity, reward sensitivity, and stress regulation could provide a more nuanced understanding of gene–behavior relationships in athletes. In addition, this study did not include direct biochemical markers of dopaminergic function, such as dopamine levels or neuroimaging indices. Incorporating such measures in future research would provide a more mechanistic understanding of the associations observed.
- Finally, the study was conducted in a Polish sample, which may limit the generalizability of the findings to other cultural or ethnic groups.

Future research combining longitudinal designs, larger multi-ethnic cohorts, and neuroimaging could clarify how dopaminergic polymorphisms interact with environmental factors to influence motivation, performance, and mental health in athletes.

## 5. Conclusions

This study examined the association between the *DRD2* Ex8 rs6276 polymorphism and personality traits in elite athletes compared with non-athlete controls. Athletes scored higher for Conscientiousness, and a genotype × group interaction was observed for Extraversion, with the main genotype effect being Agreeableness. These findings are partially consistent with the initial hypothesis: rs6276 was associated with Extraversion (genotype × group interaction), while for Conscientiousness, the effect reflected group status rather than genotype.

The results suggest that dopaminergic genetic variation may contribute to individual differences in social and motivational traits that are particularly relevant for adaptation to the demands of elite sport. However, the effects observed in this study accounted for only a small proportion of variance and should therefore be interpreted with caution.

The findings support a gene × environment interaction framework, indicating that genetic influences on personality become most evident in structured, high-demand environments such as elite athletics. At the same time, personality traits are polygenic and shaped by multiple biological and environmental factors; future studies should therefore employ polygenic approaches, include complementary psychological measures, and use longitudinal designs to better understand how dopaminergic variability contributes to motivation, adaptation, and mental health in athletes.

## Figures and Tables

**Table 1:** Hardy–Weinberg equilibrium analysis for athletes and control participants.

Genotypes		Observed (Expected)	Allele Freq	χ^2^ (*p* Value)
Athletes n = 141	A/G	50 (66.6)	p (A) = 0.62 q (G) = 0.38	8.7900 (0.0030)
	A/A	62 (53.7)		
	G/G	29 (20.7)		
Control n = 182	A/G	82 (84.7)	p (A) = 0.63 q (G) = 0.37	0.1810 (0.6704)
	A/A	74 (72.7)		
	G/G	26 (24.7)		

*p*—statistical significance χ^2^ test.

**Table 2:** Genotype and allele frequencies of *DRD2* Ex8 rs6276 in athletes and controls.

*DRD2* Ex8 rs6276					
	Genotypes			Alleles	
	A/G n (%)	A/A n (%)	G/G n (%)	A n (%)	G n (%)
Athletes n = 141	50 (35.46%)	62 (43.97%)	29 (20.57%)	174 (61.70%)	108 (38.30%)
Control n = 182	82 (45.05%)	74 (40.66%)	26 (14.29%)	230 (63.19%)	134 (36.81%)
χ^2^ (*p* value)	3.838 0.1468			0.1495 (0.6990)	

n—number of subjects.

**Table 3:** NEO-FFI sten scores in athletes and healthy controls.

NEO-FFI	Athletes (n = 141)	Control (n = 182)	Z	(*p*-Value)
Neuroticism	4.86 ± 2.15	4.48 ± 1.96	1.296	0.1951
Extraversion	6.49 ± 1.85	6.63 ± 1.77	−0.8259	0.4088
Openness	4.84 ± 2.35	4.54 ± 1.62	1.0890	0.2761
Agreeableness	5.96 ± 3.94	5.77 ± 1.94	−0.1147	0.9087
Conscientiousness	7.08 ± 2.07	6.10 ± 2.10	4.0314	0.0001 *#

*p*—statistical significance with Mann–Whitney U-test; n—number of subjects; M ± SD, mean ± standard deviation; * statistically significant differences; # Bonferroni correction was applied, and the *p*-value was lowered to 0.01 (*p* = 0.05/5 (number of statistical tests performed)).

**Table 4:** Associations between *DRD2* Ex8 rs6276 genotypes and NEO-FFI personality dimensions in athletes and controls.

NEO-FFI	Group	GENOTYPES				ANOVA		
		A/G n = 132 M ± SD	A/A n = 136 M ± SD	G/G n = 55 M ± SD	Factor	F (*p* Value)	η^2^	Power (Alfa = 0.05)
Neuroticism	Athletes; n = 141	4.88 ± 2.153	5.00 ± 2.10	4.52 ± 2.29	intercept Athletes/Control *DRD2* Ath./Control × *DRD2*	F_1,317_ = 1366.88 (*p* < 0.0001) *# F_1,317_ = 1.69 (*p =* 0.1942) * F_2,317_ = 0.89 (*p* = 0.4121) F_2,307_ = 0.32 (*p* = 0.7266)	0.812 0.005 0.006 0.002	1.000 0.254 0.203 0.101
	Control; n = 182	4.29 ± 1.95	4.70 ± 1.98	4.42 ± 1.90				
Extraversion	Athletes; n = 141	6.20 ± 1.56	6.48 ± 1.83	7.00 ± 2.27	intercept Athletes/Control *DRD2* Ath./Control × *DRD2*	F_1,317_ = 3547.58 (*p* < 0.0001) *# F_1,317_ = 0.16 (*p =* 0.6906) F_2,317_ = 0.16 (*p* = 0.8567) F_2,317_ = 5.13 (*p* = 0.0064) *#	0.918 0.0005 0.001 0.031	1.000 0.068 0.074 0.822
	Control; n = 182	6.99 ± 1.98	6.47 ± 1.48	5.96 ± 1.66				
Openness	Athletes; n = 141	4.64 ± 1.75	4.71 ± 1.90	5.45 ± 3.74	intercept Athletes/Control *DRD2* Ath./Control × *DRD2*	F_1,317_ = 540.30 (*p* < 0.0001) *# F_1,317_ = 2.76 (*p =* 0.0976) F_2,317_ = 1.11 (*p* = 0.3305) F_2,317_ = 1.25 (*p* = 0.2870)	0.829 0.009 0.007 0.008	1.000 0.381 0.245 0.272
	Control; n = 182	4.72 ± 1.53	4.34 ± 1.64	4.54 ± 1.84				
Agree-ableness	Athletes; n = 141	5.58 ± 2.00	5.68 ± 2.47	7.21 ± 1.43	intercept Athletes/Control *DRD2* Ath./Control × *DRD2*	F_1,317_ = 1111.59 (*p* < 0.0001) * F_1,317_ = 0.40 (*p =* 0.5298) F_2,317_ = 3.88 (*p* = 0.0216) * F_2,317_ = 0.46 (*p* = 0.6332)	0.778 0.001 0.024 0.003	1.000 0.096 0.699 0.124
	Control; n = 182	5.78 ± 1.88	5.50 ± 2.03	6.50 ± 1.77				
Conscientiousness	Athletes; n = 141	6.86 ± 2.05	7.24 ± 2.09	7.11 ± 2.08	intercept Athletes/Control *DRD2* Ath./Control × *DRD2*	F_1,317_ = 2582.96 (*p* < 0.0001) *# F_1,317_ = 16.00 (*p =* 0.0001) *# F_2,317_ = 0.45 (*p* = 0.6383) F_2,317_ = 0.41 (*p* = 0.6605)	0.891 0.048 0.003 0.003	1.000 0.979 0.123 0.117
	Control; n = 182	6.13 ± 2.08	6.18 ± 2.19	5.81 ± 1.98				

Ath.—athletes; *—significant result; M ± SD—mean ± standard deviation; n—number of subjects; *p*—statistical significance (ANOVA test); η^2^—effect size (partial eta squared); # Bonferroni correction was applied, and the *p*-value was lowered to 0.01 (*p* = 0.05/5 (number of statistical tests per-formed)).

**Table 5:** Post hoc analysis (Least Significant Difference test) of *DRD2* Ex8 rs6276 × group interactions for Extraversion, Agreeableness, and Conscientiousness (sten scores).

Genotypes and Extraversion						
	{1} M = 6.20	{2} M = 6.48	{3} M = 7.00	{4} M = 6.99	{5} M = 6.47	{6} M = 5.96
Athletes A/G {1}		0.4043	0.0562	0.0146 *	0.4050	0.5817
Athletes A/A {2}			0.2005	0.0951	0.9718	0.2122
Athletes G/G {3}				0.9748	0.1795	0.0323 *
Control A/G {4}					0.0735	0.0112 *
Control A/A {5}						0.2106
Control G/G {6}						
**Genotypes and Agreeableness**						
	{1} M = 5.58	{2} M = 5.68	{3} M = 7.21	{4} M = 5.78	{5} M = 5.50	{6} M = 6.50
Athletes A/G {1}		0.8627	0.0192 *	0.7062	0.8828	0.1997
Athletes A/A {2}			0.0223 *	0.8363	0.7281	0.2353
Athletes G/G {3}				0.0265 *	0.0089 *	0.3774
Control A/G {4}					0.5551	0.2811
Control A/A {5}						0.1395
Control G/G {6}						
**Genotypes and Conscientiousness**						
	{1} M = 6.68	{2} M = 7.25	{3} M = 7.11	{4} M = 6.13	{5} M = 6.18	{6} M = 5.81
Athletes A/G {1}		0.3353	0.6178	0.0545	0.0755	0.0387 *
Athletes A/A {2}			0.7720	0.0019 *	0.0034 *	0.0036 *
Athletes G/G {3}				0.0347 *	0.0461 *	0.0235 *
Control A/G {4}					0.9017	0.4895
Control A/A {5}						0.4419
Control G/G {6}						

*—significant statistical differences; M—mean; {1} Athletes A/G; {2} Athletes A/A; {3} Athletes G/G; {4} Control A/G; {5} Control A/A; {6} Control G/G.

## Data Availability

The data presented in this study are available on request from the corresponding author. The data are not publicly available due to privacy concerns.

## Supplementary Materials

The following supporting information can be downloaded at https://www.mdpi.com/article/10.3390/brainsci15090965/s1, Table S1: Genetic association of polymorphisms of the *DRD2* Ex8 rs6276 in athletes and controls; Figure S1: Interaction between group status (athletes vs. controls) and *DRD2* Ex8 rs6276 genotypes on Extraversion (NEO-FFI sten scores), presenting a line plot with standard errors (SEs). The supplementary figure is provided to illustrate the interaction pattern underlying the results presented in the main text (Figure 1).
