# Supplementary material for: DRD2 Ex8 rs6276 Polymorphism and NEO-FFI Personality Traits in Elite Athletes and Controls
Source: Brain Sci. 2025 Sep 5;15(9):965. doi: 10.3390/brainsci15090965 (PMC12467987; doi:10.3390/brainsci15090965)
Supplement: Supplementary file 1 [file brainsci-15-00965-s001.zip › brainsci-3848118-supplementary-table.pdf]

Supplementary Table S1. Genetic association of polymorphisms of the *DRD2* Ex8 rs6276 in athletes and controls.

Model	Genotype	Athletes n = 141	Control n = 182	OR (95% CI)	*p*-value
CD	A/G	50 (35.46)	82 (45.05)	1.00	
	A/A	62 (43.97)	74 (40.65)	0.73 (0.45-1.18)	0.20079
	G/G	29 (20.57)	26 (14.29)	0.54 (0.29-1.03)	0.06106
Dom	G/G	29 (20.57)	26 (14.29)	1.00	
	**A/A + A/G**	112 (79.43)	156 (85.71)	1.55 (0.87-2.78)	0.13633
Rec	A/A	62 (43.97)	74 (40.66)	1.00	
	**G/G + A/G-**	79 (56.02)	108 (59.34)	1.14 (0.73-1.79)	0.54986
OD	A/A + G/G	91 (64.54)	100 (54.95)	1.00	
	**A/G**	50 (35.46)	82 (45.05)	0.67 (0.43-1.05)	0.08194

CD: codominant; Dom: dominant; OD: overdominant; Rec: recessive.
