# Supplementary figures and images for: DRD2 Ex8 rs6276 Polymorphism and NEO-FFI Personality Traits in Elite Athletes and Controls
Source: Brain Sci. 2025 Sep 5;15(9):965. doi: 10.3390/brainsci15090965 (PMC12467987; doi:10.3390/brainsci15090965)

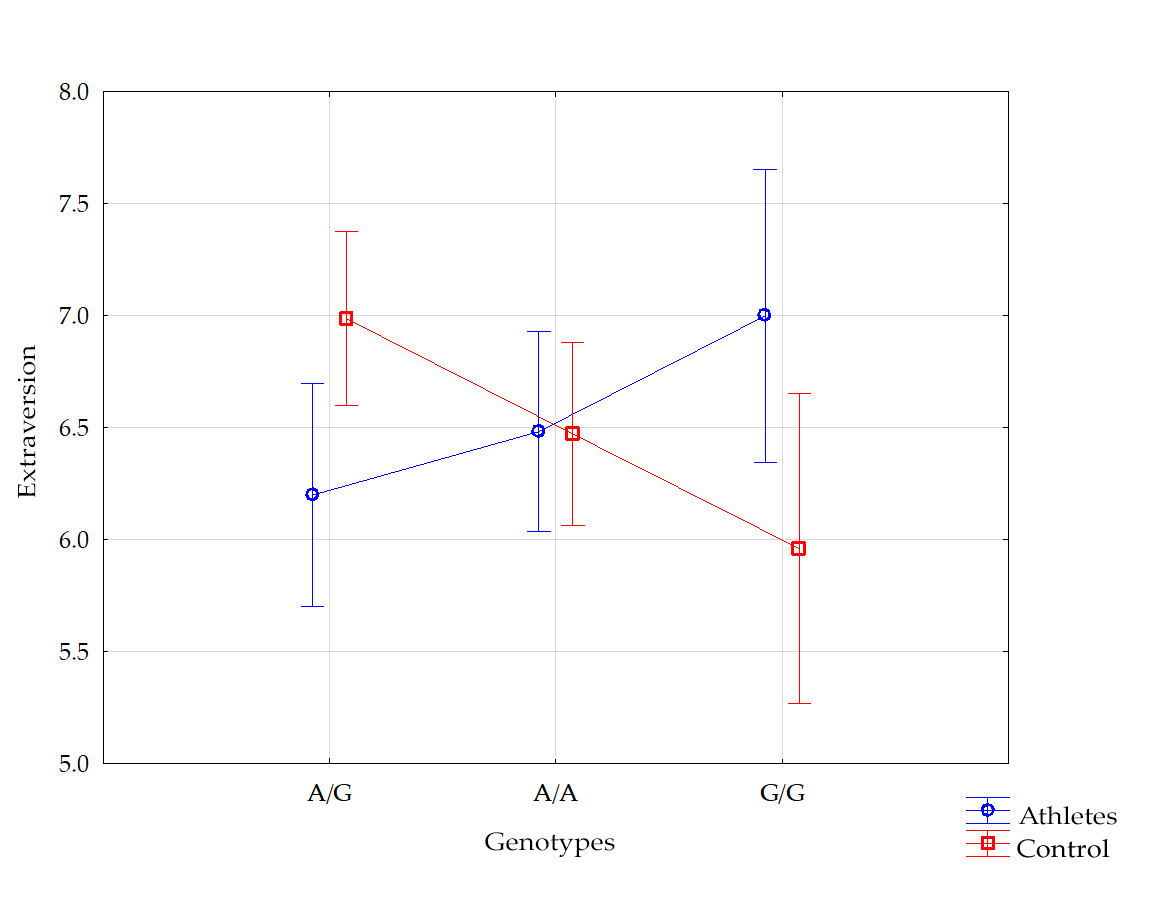

Supplement: Supplementary file 1 [file brainsci-15-00965-s001.zip › brainsci-3848118-supplementary-figure.jpg]
